# Reliable aortic arch measurements using a novel cardiac magnetic resonance sequence: navigated 3D SPACE

**DOI:** 10.1186/1532-429X-18-S1-P160

**Published:** 2016-01-27

**Authors:** Hari K Narayan, Yoav Dori, Matthew A Harris, Marc S Keller, Gary R McNeal, Mark A Fogel, Kevin K Whitehead

**Affiliations:** 1 Department of Pediatrics, The Children's Hospital of Philadelphia, Philadelphia, PA USA; 2 Department of Radiology, The Children's Hospital of Philadelphia, Philadelphia, PA USA; 3 Siemen's Healthcare, Malvern, PA USA

## Background

Existing cardiac magnetic resonance sequences are frequently suboptimal in the measurement of the aortic arch, particularly in the case of arch obstruction. Navigated three-dimensional (3-D) sampling perfection with application optimized contrast using different flip angle evolution (SPACE) is a novel turbo spin echo dark blood cardiac magnetic resonance sequence that yields a robust 3-D dataset and gates in systole in order to demonstrate the maximal aortic caliber. Additionally, it utilizes susceptibility for optimal blood suppression by orienting blood flow in the readout direction, thereby enhancing imaging in the setting of turbulence. We report our experience measuring the aortic arch using this sequence.

## Methods

Patients with congenital heart disease who were scheduled to undergo clinically indicated cardiac magnetic resonance imaging were prospectively enrolled and underwent navigated 3-D SPACE imaging. Aortic arch measurements were obtained from navigated 3-D SPACE images and other magnetic resonance sequences that were performed during the same study. Absolute agreement mixed-effects intraclass correlation coefficients (ICC) were used to determine agreement between navigated 3-D SPACE and other commonly used sequences as measured by a single blinded investigator and agreement between two blinded investigators in a randomly selected sub-sample of the navigated 3-D SPACE studies.

## Results

Analysis was performed on 43 studies. The ICC for inter-observer agreement in 13 navigated 3-D SPACE sequences was 0.973 (p < 0.001). The ICCs for agreement between measurements derived from navigated 3D SPACE and those derived from steady state free precession Cine, two-dimensional dark blood imaging and gadolinium-enhanced magnetic resonance angiography were 0.734 (p < 0.001), 0.891 (p < 0.001) and 0.951 (p < 0.001) respectively.

## Conclusions

Navigated 3-D SPACE aortic arch measurements demonstrated excellent reliability between observers and validity in comparison to existing cardiac magnetic resonance sequences in patients with congenital heart disease. Given that the sequence's attributes are uniquely suited to measuring arch obstruction, it may be a useful tool in cases where accurate measurements of the aortic arch are clinically important

**Figure 1:**
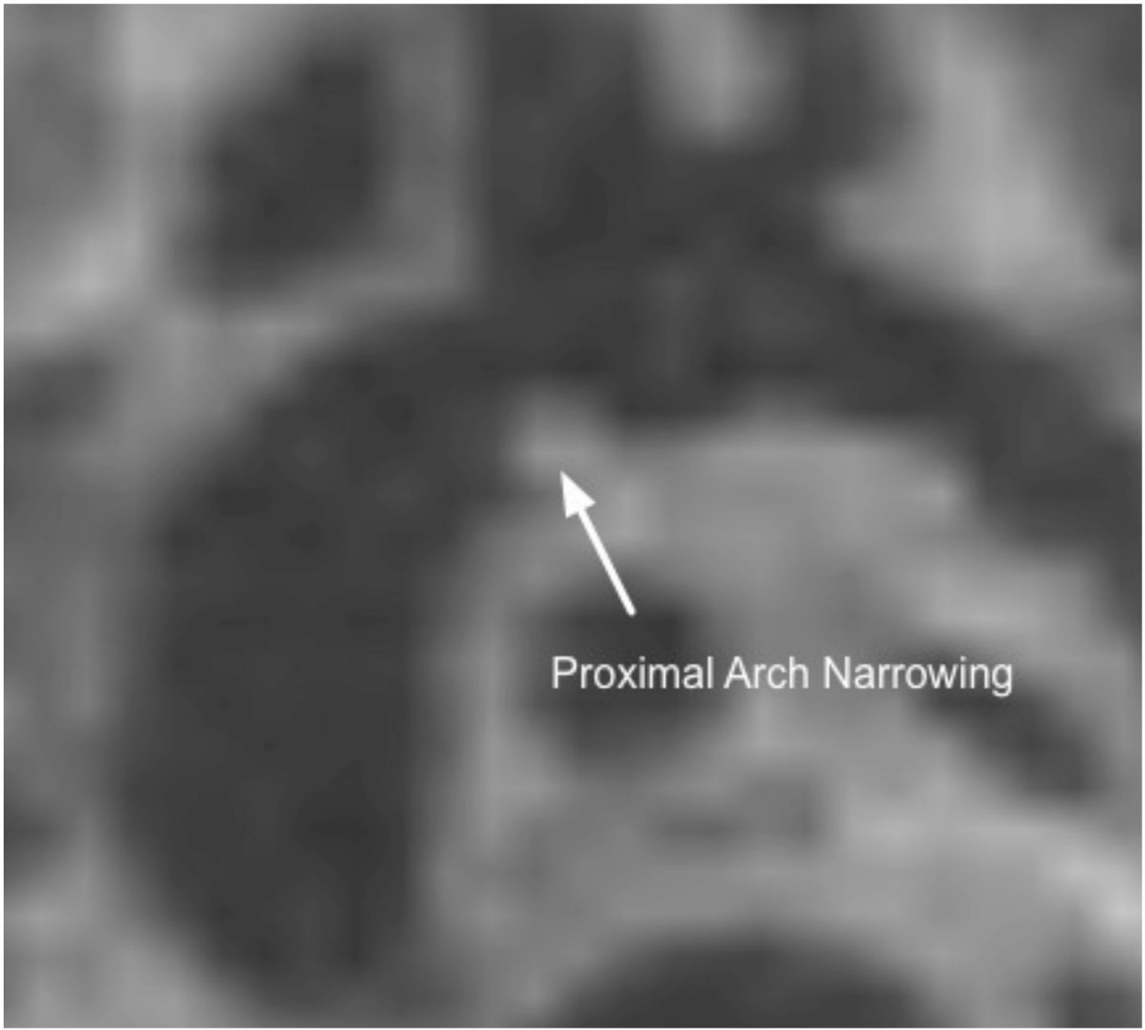
**Navigated 3D SPACE image demonstrated discrete proximal aortic arch narrowing in a 6 month old with a history of arch hypoplasia and coarctation of the aorta who previously underwent an arch augmentation**.

